# Disparities in Hepatitis C Linkage to Care in the Direct Acting Antiviral Era: Findings From a Referral Clinic With an Embedded Nurse Navigator Model

**DOI:** 10.3389/fpubh.2019.00362

**Published:** 2019-11-27

**Authors:** Jacqueline E. Sherbuk, Kathleen A. McManus, Terry Kemp Knick, Chelsea E. Canan, Tabor Flickinger, Rebecca Dillingham

**Affiliations:** ^1^Division of Infectious Diseases and International Health, University of Virginia, Charlottesville, VA, United States; ^2^Department of Medicine, University of Virginia, Charlottesville, VA, United States

**Keywords:** hepatitis C, linkage to care, cascade of care, health disparities, substance use disorder

## Abstract

**Background:** Direct acting antivirals (DAAs) have simplified and expanded access to Hepatitis C virus (HCV) treatment. Only 17% of the 2.4 million Americans with HCV have linked to HCV care. We aimed to evaluate linkage to care (LTC) in a non-urban HCV referral clinic with a nurse navigator model and identify disparities in LTC.

**Methods:** A single-center retrospective cohort analysis was performed among all patients referred to an infectious diseases HCV clinic between 2014 and 2018. The primary outcome was LTC, defined as attendance at a clinic appointment. A multivariable Poisson regression model estimated the association of variables with LTC.

**Results:** Among 824 referred patients, 624 (76%) successfully linked to care and 369 (45%) successfully achieved sustained virologic response. Forty-six percent of those referred were uninsured. On multivariable analysis, LTC rates were higher among women (Incidence Rate Ratio [IRR] 1.11, 95% CI 1.03–1.20, *p*-value = 0.01) and people with cirrhosis (IRR 1.20, 95% CI 1.11–1.30, *p*-value < 0.001). Lower LTC rates were found for young people (<40 years; IRR 0.88, 95% CI 0.79–0.98, *p*-value = 0.02) and uninsured people (IRR 0.85, 95% CI 0.77–0.94, *p*-value = 0.002). Among those without LTC, 10% were incarcerated. Race, proximity to care, substance use, and HIV status were not associated with LTC.

**Conclusions:** Using an embedded nurse navigator model, high LTC rates were achieved despite the prevalence of barriers, including a high uninsured rate. Disparities in LTC based on age, sex, and insurance status are present. Substance use was not associated with LTC. Future interventions to improve care should include expanded access to insurance and programs bridging care for incarcerated populations.

## Introduction

The Centers for Disease Control and Prevention estimates that 2.4 million Americans are currently infected with hepatitis C virus (HCV) (1). The development of effective and well-tolerated direct acting antiviral (DAA) therapy has made the goal of HCV elimination possible (2, 3). For those living with HCV, achieving cure is cost-effective and is associated with decreased complications of HCV and improvement in patient related outcomes (4).

The HCV cascade of care defines the steps required to progress from diagnosis to cure and is generally structured around the steps of testing, linkage to care (LTC), and treatment, though the exact outcomes measured vary by study (5). The LTC step bridges individuals from diagnosis with HCV, often identified through public health and screening programs, to treatment, usually initiated at medical clinics. While guidelines recommend that all people with active HCV be linked to a clinician (6), LTC is frequently shown to be a point in the cascade where there is a large drop-off (5, 7–10). Unfortunately, only 17% of those confirmed to have HCV have been linked to specialty care nationwide (10) and continuing the current approach to care is unlikely to lead to elimination (4). A paucity of data exists regarding LTC compared to that regarding the testing and treatment steps of the cascade, and compounding this lack of data, most LTC studies are from the pre-DAA era (5).

Access to HCV treatment is expanding as infectious disease specialists, general practitioners, and primary care nurse practitioners join gastroenterologists and hepatologists to provide HCV treatment. Unfortunately, access to HCV care and treatment remains unequal. Disparities in LTC have been identified for racial minorities, those lacking private insurance, rural populations, and those who use substances (11–13). Further interventions are needed to improve LTC rates and to identify and mitigate disparities. The development of telehealth models connecting primary care providers with subspecialists (14) and the use of patient navigators or care coordinators (15, 16) are models of care aimed to improve access and LTC, respectively. Our HCV referral clinic utilizes a nurse navigator model of care. In this model, the nurse coordinator provides individualized patient-centered care by educating patients on HCV and available resources, connecting patients to care, and supporting patients through the treatment process.

Given the paucity of data on the critical LTC step of the cascade, a deeper understanding of the current state of LTC is needed. We aimed to evaluate disparities in LTC in the current DAA era among all individuals referred to an infectious diseases HCV clinic with a low barrier to entry and a nurse navigator model.

## Methods

### Study Design

A single-center retrospective cohort study was performed. The study population was defined to be all adults age 18 years or older referred to the University of Virginia (UVA) Infectious Diseases HCV clinic from November 2014 through March 2018. Referred individuals who could not be identified within the UVA electronic medical record were excluded. This study was approved by the UVA Health Sciences Research Institutional Review Board.

### Clinical Setting

UVA is a tertiary care referral center that acts as a safety net hospital. Financial assistance is available and patients are able to obtain necessary labs, imaging, and visits with specialty physicians. The UVA Infectious Diseases HCV clinic is co-located within the Ryan White HIV/AIDS Program-funded clinic. The HCV clinic staff includes physicians, a full time nurse coordinator, and a pharmacy based team. The clinic is designed to have a low barrier to entry. Patients can be referred through internal referrals from within the UVA health system, external referrals including community providers and local health departments, and by self-referral. Upon referral, the nurse coordinator directly contacts the referred patient by phone to schedule an appointment. The nurse coordinator will make multiple attempts to reach patients if calls are unanswered and appointments are rescheduled in the case of no-shows. Referred patients are also provided the contact information for the clinic at the time of referral. The nurse coordinator provides education and counseling, both over the phone and at clinic appointments, as well as ensuring appropriate paperwork is completed for medication approval. Medications may be obtained through private insurance, government insurance, or pharmaceutical patient assistance programs for uninsured patients. The nurse navigator assists patients in completing required financial screening, prior authorizations, and patient assistance program paperwork. Fibroscans are performed within the clinic for staging of liver disease. A pharmacy-based team also assists with prior authorizations and provides telephone-based counseling related to HCV medications as well as potential interactions with patients' other medications.

### Sample

During the study time period, 834 patients were referred to the clinic. Of these, 10 patients referred from outside of the health system could not be identified in the electronic medical record and were therefore excluded. A total of 824 patients were included in analyses.

### Data Collection

Referred patients were identified through a clinic database. The database, managed by the nurse coordinator, is used to track patients' progression along the cascade of care. Data on completion of cascade steps, free text reason for failure to link to care, and source of referral (internal vs. external) were obtained from this database. The UVA Clinical Database Repository was used to identify independent variables, demographic and clinical characteristics of study participants including age, sex, race, insurance status, residence location, and HCV genotype. Patients were determined to live in “close proximity to UVA” if residence was in the city of Charlottesville or surrounding counties. The clinical database repository also identified the presence of comorbidities including cirrhosis, HIV, hepatitis B, and substance use based on ICD9/ICD10 codes.

### Outcomes

The primary outcome and dependent variable for this study was LTC, defined to be attendance at an HCV clinic appointment. Secondary outcomes included the remaining steps in the cascade of care. Cascade steps were defined to be: (1) Referral, (2) LTC, (3) Medication prescribed by the specialist physician, (4) Medication initiated per patient report, (5) Treatment course completed per patient report and pharmacy records, (6) Post-treatment viral load measured 12 weeks after completing therapy, and (7) Sustained virologic response (SVR) achieved, defined as an undetectable viral load 12 weeks after completing therapy.

### Statistical Analysis

Univariate and multivariate analyses were performed comparing clinical and demographic characteristics among those who successfully linked to care and those who did not. For univariate analyses, chi-square analysis or Fisher's exact, if applicable, was performed for categorical variables and a Student's *T*-test was performed for continuous variables. A multivariate Poisson regression model estimated associations of patient characteristics with LTC. The multivariate model included demographic characteristics (age, sex, race), insurance status, proximity to care, and medical comorbidities. Age, sex, race, and insurance status were included in the model, as these variables have previously been associated with progression along the cascade (10, 13, 15, 17–21). Proximity to care and comorbidities were included because they may influence the referred patients' relationship with the clinic in the case of those living in close proximity or those living with HIV, as well as potentially impacting treatment decisions in the case of cirrhosis, hepatitis B, and HIV. To address the potential issue of overdisperson in Poisson regression, we ran a negative binomial model which demonstrated that the data is not overdispersed. Therefore, the Poisson model is appropriate for our data. Statistical analyses were performed using Stata 15.0 (StataCorp LLC, College Station, TX). A *p*-value < 0.05 was determined to represent significance.

## Results

**Figure 1:**
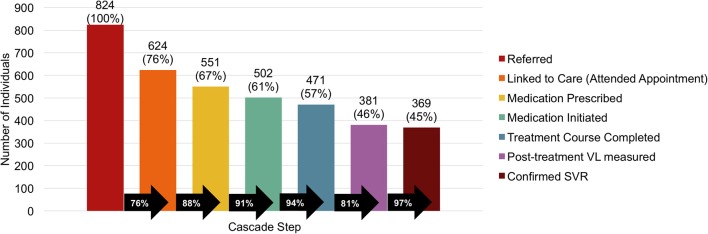
Hepatitis C cascade of care. VL, viral load; SVR, sustained virologic response.

Successful LTC occurred in 624 (76%) of 824 referred patients (Figure 1). Of those referred, medication was prescribed for 551 (67%), initiated in 502 (61%), and completed by 471 (57%). A post-treatment viral load was measured in 381 (46%) and SVR was confirmed in 369 (45%) of those referred. The largest drop-offs in the cascade of care were 24% failing to link to care and 19% failing to have a post-treatment viral load.

Referred patients were predominately white (67%) and male (57%) with a mean age was 48.5 years (Table 1). Nearly half of those referred were uninsured (46%), and a minority lived in close proximity to the referral clinic (27%). Only 8% were co-infected with HIV. Rates of cirrhosis and hepatitis B were low at 10 and 3%, respectively. Substance use was present in 22%. The source of referral was available for 470 individuals: 43% internal referrals and 57% external referrals. Of those with HCV genotype data available, 470 of 537 (88%) had genotype 1.

**Table 1:** Baseline characteristics of referred patients.

	**All**	**Failure in LTC**	**Successful LTC**	***P*-value**
	***N* = 824**	***N* = 200**	***N* = 624**	
	***N* (%)**	***N* (%)**	***N* (%)**	
Age (years)				
Mean (SD)	48.5 (13.5)	45.1 (13.9)	49.6 (13.2)	<0.0001
Age (by decade)				0.003
18–29	105 (13)	35 (18)	70 (11)	
30–39	137 (17)	45 (23)	92 (15)	
40–49	112 (14)	25 (13)	87 (14)	
50–59	282 (34)	62 (31)	220 (35)	
60+	188 (23)	33 (17)	155 (25)	
Sex				0.22
Male	468 (57)	121 (61)	347 (56)	
Female	356 (43)	79 (40)	277 (44)	
Race				0.005
White	551 (67)	137 (69)	414 (66)	
Black	182 (22)	34 (17)	148 (24)	
Other	61 (7)	14 (7)	47 (8)	
Unknown	30 (4)	15 (8)	15 (2)	
Insurance				0.001
Private	127 (15)	21 (11)	106 (17)	
Medicaid	182 (22)	39 (20)	143 (23)	
Medicare	133 (16)	23 (12)	110 (18)	
Uninsured	382 (46)	117 (59)	265 (42)	
Close proximity	221 (27)	55 (28)	166 (27)	0.87
Comorbidities				
Cirrhosis	84 (10)	6 (3)	80 (13)	<0.001
HIV	67 (8)	11 (6)	56 (9)	0.12
Hepatitis B	22 (3)	1 (1)	21 (3)	0.07
Substance Use	184 (22)	41 (21)	143 (23)	0.48
Referral Source^a^				0.11
Internal	204 (43)	66 (44)	138 (43)	
Community Providers	144 (31)	38 (25)	106 (33)	
Health Department	76 (16)	33 (22)	43 (13)	
Self/Family	39 (8)	11 (7)	28 (9)	
Free Clinic	7 (2)	3 (2)	4 (1)	
Genotype^b^				0.07
1	470 (88)	37 (77)	433 (89)	
2	31 (6)	7 (15)	24 (5)	
3	34 (6)	4 (8)	30 (6)	
4	1 (0.2)	0 (0)	1 (0.2)	
6	1 (0.2)	0 (0)	1 (0.2)	

a *Referral data available for N = 470 individuals*.

b *Genotype data available for N = 537 individuals*.

*LTC, Linkage to Care; HIV, human Immunodeficiency Virus*.

*Statistical analyses for categorical variable included chi-square analysis or Fisher's exact, if a value <5 was included in that analysis. For mean age, Student's T-test was performed*.

Among those who failed to link to care, the most common reasons documented by the nurse navigator for failure to link to care included patients failing to present to clinic despite having multiple appointments scheduled (26.5%) and inability to contact patient to schedule an appointment (20.5%). Incarceration prevented LTC in 10% (Table 2).

**Table 2:** Reasons for failure to link to care at our referral clinic (*N* = 200).

**Reason**	**N**	**%**
Multiple no-shows despite scheduled appointments	53	26.5%
Unable to contact to schedule an appointment	41	20.5%
No reason documented	24	12.0%
Incarcerated	20	10.0%
Patient Preference	19	9.5%
Referred/Treated Elsewhere	16	8.0%
Moved Out of Area	10	5.0%
Deceased	5	2.5%
Pregnant	4	2.0%
Spontaneous Viral Clearance	4	2.0%
Other^a^	4	2.0%

*Data obtained from clinic database maintained by nurse coordinator*.

a *“Other” includes lack of transportation (1), work conflict with clinic schedule (1), initial visit scheduled and upcoming (1), deferred due to upcoming surgery (1)*.

Univariate analysis showed that those who successfully linked to care were older (*p*-value < 0.0001) (Table 1). Data on race was missing in 15 individuals (8%) who failed to link to care and 15 (2%) who successfully linked to care. Racial differences were present (*p*-value = 0.005) with black individuals having higher rates of LTC. Those successfully linking to care were more likely to have Medicare, Medicaid, and private insurance while being uninsured was more common in those failing to link to care (59 vs. 42%). The proportion of individuals with cirrhosis was higher in those successfully linking to care (13 vs. 3%, *p*-value < 0.001). Sex, proximity to care, HIV status, and substance use did not differ between groups.

The multivariate Poisson regression analysis identified higher rates of LTC among women (Incidence Rate Ratio [IRR] 1.11, 95% Confidence Interval [CI] 1.03-1.20, *p*-value = 0.007), those with cirrhosis (IRR 1.20, 95% CI 1.11–1.30, *p*-value < 0.001), and those co-infected with hepatitis B (IRR 1.18, 95% CI 1.06–1.32, *p*-value = 0.002) (Table 3). Young people, defined to be < 40 years of age, had lower rates of LTC (IRR 0.88, 95% CI 0.79–0.98, *p*-value = 0.02), as did those who are uninsured (IRR 0.85, 95% CI 0.77–0.94, *p*-value = 0.002). Race, proximity to care, HIV status, and substance use were not associated with rates of LTC.

**Table 3:** Patient characteristics associated with successful linkage to care in a multivariable Poisson regression model (*N* = 762).

**Patient Characteristic**	**IRR (95% CI)**	***P*-value**
Age		
>40 years	1 (ref)	na
≤ 40 years	0.88 (0.79–0.98)	0.02
Sex		
Men	1 (ref)	na
Women	1.11 (1.03–1.20)	0.007
Race		
White	1 (ref)	na
Black	1.07 (0.97–1.18)	0.17
Other	1.06 (0.93–1.21)	0.41
Insurance Coverage		
Private	1 (ref)	na
Medicaid/Medicare	0.91 (0.83–1.01)	0.07
Uninsured	0.85 (0.77–0.94)	0.002
Close Proximity to medical center	0.93 (0.85–1.02)	0.13
Cirrhosis	1.19 (1.10–1.30)	<0.001
Hepatitis B	1.18 (1.06–1.32)	0.002
HIV	1.02 (0.90–1.15)	0.78
Substance Use	0.99 (0.90–1.10)	0.90

*Incidence rate ratios are adjusted for the variables included in the table*.

*IRR, Incidence Rate Ratio; CI, Confidence Interval; HIV, Human Immunodeficiency Virus*.

## Discussion

In this cohort referred to an infectious diseases HCV clinic, the overall rate of LTC was high despite the presence of barriers to care including a large uninsured population. We identified disparities in LTC among men, uninsured patients, and young people. While co-located within a Ryan White HIV/AIDS Program clinic, our population had a low rate of HIV co-infection compared to other infectious diseases HCV clinics (22).

Our LTC rate of 76% was comparable or higher than reported in other studies, though comparison of LTC rates across studies is challenged by the lack of a standard definition of LTC (5). Nationally, a surveillance study reported a 17% LTC rate among those confirmed to have a positive viral load in 2016 (10). Reported rates of LTC are higher within the Veterans Affairs medical system, where 56% of those diagnosed were evaluated in specialty clinics (23), and highly variable in studies within a single health system, ranging from 23% (24) to 82% (12).

We attribute our high rate of LTC to a proactive nurse navigator model of care. Engaging patients in care is time intensive, and investment in a full time nurse coordinator is a critical component of our model. Our nurse clinic coordinator actively attempts to contact referred patients by phone and letter, including continued attempts to contact those lost to follow-up. The coordinator provides education and counseling over the phone and at the patient visits and completes required paperwork for medication approval. Prior authorizations and patient assistance paperwork are labor intensive, as they vary by insurance plans and pharmaceutical company. Requirements also shift frequently. Dedicated staffing is critical for timely acquisition of medication. Through the dedicated nurse coordinator and the pharmacy-based team, our model was successful in obtaining treatment for those who linked to care through either patients' insurance coverage, with copay assistance as needed, or patient assistance programs. Within our clinic we were able to obtain affordable treatment for all patients who were eligible.

Interestingly, a few recent reports have suggested a slight decrease in LTC rates since the initial introduction of DAAs (10, 12). This has been attributed to having a large pool of motivated individuals at the time DAAs came to market. Improving, or even maintaining, current LTC rates will require enhanced screening and linkage programs. Peer-based programs (25), patient navigators, and case managers have been shown to be helpful in linking patients to care (16, 26), though successful treatment rates following LTC may still remain low (26). In an aggressive outreach campaign for high risk veterans with HCV through the VA, despite repeated attempts to engage veterans, 30% were unable to engage in care (27). Telehealth, such as the ECHO model and collaborations between HCV specialists and general practitioners have expanded access to treatment, with a focus on rural regions (14). For those with a history of substance use, linkage to a clinic with embedded support services improves outcomes (28).

While our LTC rate was high, the LTC step was also the largest drop-off within our cascade. In accordance with prior studies, we identified disparities in LTC, with lower rates of LTC in men (18–20) and among young people (10, 17–19). This is concerning given that highest rate of ongoing transmission occurs in younger individuals (29). Treatment of this population yields not only individual benefits but also broader public health benefits including decreased risk of ongoing transmission. Co-infection with HBV and the presence of cirrhosis were associated with higher rates of LTC, consistent with prior studies (20, 23), which may be due to increased patient motivation in the presence of greater perceived risk of disease progression. Race, proximity to care, and substance use were not associated with LTC rates. Substance use has previously and in some cases remains a barrier to treatment through insurance and Medicaid restrictions (30). Providers may also consider substance use to be a contraindication to treatment (31) and be reluctant to prescribe treatment despite guidelines recommending treatment regardless of substance use status (6). Our findings that people with substance use link to care at similar rates as those without a history of substance use support prior studies demonstrating that people with a history of substance use can successfully adhere to treatment and achieve high rates of cure (32).

Nearly half of referred patients were uninsured. Once linked to care, our team has been able to obtain treatment through patient assistance programs for those uninsured. Unfortunately, those lacking insurance may not have been aware of these alternative options and may have been less likely to link to care, in accordance with prior studies (13, 15, 20). Expanding access to insurance, such as through Medicaid expansion, may help mitigate this barrier to care. As of January 1, 2019, Virginia has expanded Medicaid, and we look forward to reporting on outcomes associated with expansion. An increase in the provider workforce will be required to meet the increased number of insured patients seeking HCV care.

The most common reasons given for failure to link to care were multiple no-shows, inability to contact patients, and incarceration. Rates of HCV in the incarcerated population are far higher than in the general population, yet only a small minority of incarcerated people have been treated for HCV (33). Treatment of people in jail is feasible (34), and treating people in the correctional system is an integral part of achieving national HCV elimination goals (6).

Despite our integrated model of care, patients continue to be lost at each step in the cascade. Simplifying the cascade would improve the proportion of patients who complete each step (35). Following LTC, the next highest drop-off in the cascade was seen in measurement of a post-treatment viral load. SVR was achieved in 97% of those who had a post-treatment viral load measure, however 19% of those who completed treatment never returned for a viral load measurement to confirm treatment success. Unfortunately, viral load testing is expensive and out of reach for many patients who may lack insurance or be under-insured. While patient assistance programs within our institution support necessary laboratory tests, including HCV viral load, this support requires that patients have their labs performed at our institution, a challenge for a medical center serving a wide geographic area where affordable transportation is unavailable for many. Medicaid expansion has the potential to mitigate this barrier through access to Medicaid transportation. Widely available and affordable testing may improve monitoring of those treated for HCV. Rapid testing is being explored as one possibility to simplify the cascade both at diagnosis and at confirmation of cure (36).

As an observational study, there are limitations to our findings. Our referred patients comprised an open cohort, and we are limited to data available through our health system's electronic medical record and our clinic database. It is possible that referred patients linked to care outside of our medical system. A number of referred patients were lost to follow-up through incarceration. Our evaluation of co-morbidities relies on the accuracy and completeness of medical providers entering ICD codes. Coding may be incomplete and may be less complete for those who failed to link to care and therefore did not attend an HCV specialist appointment where diagnoses could have been coded. We are unable to account for referral source or genotype in our multivariable analysis due to limited available data.

Linkage to care is a crucial step connecting those diagnosed to treatment. As treatment restrictions have loosened and access to treatment has expanded, successfully linking diagnosed patients to an appointment for HCV care is critical. Approaches and interventions to improve LTC rates should directly address men, young people, and the uninsured who are less likely to link to care. Further exploration of those who do not link to care through a qualitative study will better define patient perceptions regarding LTC. Next steps will also include investigation of interventions that could decrease no show rates and improve patient-clinic communication, such as mobile health technology. Use of a nurse-navigator model successfully overcame some previously reported barriers such as proximity to care, substance use, and race.

## Conclusions

Our nurse-navigator model of care achieved high rates of LTC, far exceeding the national average, though disparities in LTC persist with regard to age, sex, and insurance status. Incarceration and inability to contact referred patients arose as barriers to care. Substance use was not associated with LTC. Our findings can guide future interventions to improve LTC with a focus on targeting young people, bridging care for incarcerated populations, and expanding access to insurance.

## Data Availability Statement

The raw data supporting the conclusions of this manuscript will be made available by the authors, without undue reservation, to any qualified researcher.

## Ethics Statement

The studies involving human participants were reviewed and approved by University of Virginia Health Sciences Research Institutional Review Board. Written informed consent for participation was not required for this study in accordance with the national legislation and the institutional requirements.

## Author Contributions

JS, KM, TK, TF, and RD contributed conception and design of the study. JS organized the database, performed data analysis, and wrote the first draft of the manuscript. All authors contributed to the manuscript revision, read, and approved the submitted version.

### Conflict of Interest

KM reports stock ownership in Gilead Sciences, Inc. RD provides consulting services for Warm Health Technologies, and mHealth company. RD was recipient of an investigator-initiated grant from Gilead Sciences, Inc. The remaining authors declare that the research was conducted in the absence of any commercial or financial relationships that could be construed as a potential conflict of interest.
